# Investigation of human tungiasis cases, Sheema District, Uganda, November 2021-February 2022

**DOI:** 10.11604/pamj.2023.46.71.41277

**Published:** 2023-10-26

**Authors:** Sherry Rita Ahirirwe, Richard Migisha, Patience Mwine, Lilian Bulage, Benon Kwesiga, Daniel Kadobera, Alex Riolexus Ario

**Affiliations:** 1 Uganda Public Health Fellowship Program, Uganda National Institute of Public Health, Kampala, Uganda

**Keywords:** Tungiasis, jiggers, outbreak, investigation, Uganda

## Abstract

**Introduction:**

no formal surveillance system exists in Uganda for jiggers (tungiasis); however, outbreaks are frequently reported in the media. On 27^th^ January 2022, a news alert reported a jiggers’ outbreak in Sheema District, Southwestern Uganda. We investigated to establish the magnitude of the problem and identify possible exposures associated with infestation to inform control measures.

**Methods:**

we defined a confirmed case as visible Tunga penetrans (T. penetrans) in the skin of a resident of any of 6 villages in Bwayegamba Parish, Sheema District, in February 2022. A suspected case was self-reported T. penetrans infestation during the three months preceding the interview. We visited all households in the 3 most affected villages in Bwayegamba Parish to identify cases and conducted interviews to identify possible exposures. We described cases by person, place, and time. We assessed socioeconomic status, household construction, mitigation measures against jiggers, and observed participants and their environments for hygiene. We conducted 2 case-control studies. One compared case-households (with ≥1 case) with control-households (without any cases). The second compared individual cases (suspected and confirmed) to neighbourhood controls.

**Results:**

among 278 households, we identified 60 case-patients, among whom 34 (57%) were male. Kiyungu West was the most affected village (attack rate=31/1,000). Cases had higher odds of being male (ORMH=2.3, 95% CI=1.3-4.0), <20 years of age (ORMH=2.0, 95%CI=1.1-3.6), unmarried (ORMH=2.97, 95% CI=1.7-5.2), unemployed (ORMH=3.28, 95% CI=1.8-5.8), and having poor personal hygiene (ORMH=3.73, 95% CI=2.0-7.4) than controls. In the household case-control study, case-households had higher odds of having dirty or littered compounds (ORMH=2.3, 95% CI=1.2-4.6) and lower odds of practicing mitigation measures against jiggers (ORMH=0.33, 95% CI=0.1-0.8) than control-households.

**Conclusion:**

males, unemployed persons, and those with poor personal or household hygiene had increased odds of tungiasis in this outbreak. Multi-sectoral, tailored interventions that improve standards of living could reduce risk of tungiasis in this area. Adding tungiasis to national surveillance reporting tools could facilitate early identification of future outbreaks.

## Introduction

Tungiasis is a parasitic skin infection due to infestation with the female sand flea *Tunga Penetrans* [1]. The female sand flea has piercing and sucking mouth parts that it uses to suck the blood and burrow into the skin of its host [2]. It thrives in sandy and dusty dry environments, such as those in sub-Saharan Africa [1]. It penetrates into skin creating a nodular swelling where it feeds on the host´s blood while growing in size. Its posterior end remains exposed to the air through which about 100-200 eggs are shed over a period of 2 weeks. Fertilized eggs once hatched develop into larvae and pupae. This stage takes about 2-3 weeks before they develop into adults capable of jumping from the ground onto the skin of the host [1-3]. Infestation is usually limited to the feet since the flea cannot jump very high [1]. However, other parts of the body, such as the hands, elbows, buttocks, and genitals, can similarly be infested, for instance, in situations where humans sleep on the floor [1,4]. Illness causes itching, oedema, swelling, pain, and desquamation at the site of infestation [4,5]. If left untreated, tungiasis can lead to other bacterial infections, tetanus, gangrene, inability to walk, and a great loss in the quality of life [4,6]. In 2011, a jiggers outbreak in Eastern Uganda was reported to affect 20,000 people, of whom 20 died [7]. In 2012, the Uganda Ministry of Health (MOH), cognisant of the disease´s burden, constituted a task force responsible for its eradication in the Eastern and North Eastern regions of the country [8]. However, little progress was made due to a lack of surveillance data on tungiasis. It is still not among the reportable neglected tropical diseases in the Health Management Information System (HMIS) tools as identified by the Sustainability Plan for Neglected Tropical Diseases (NTDs) Control Program 2020-2025 [9]. The disease continues to spread in rural communities in Uganda due to a number of individual factors, such as high levels of poverty and environmental factors, such as presence of animal reservoirs for the flea [1,10]. On 27^th^ January 2022, a jiggers´ outbreak was reported in Sheema District, located in South Western Uganda. The news alert indicated that 80 families in 5 villages in Bwayegamba Parish, Kigarama Sub-county were affected [11]. We investigated to establish the magnitude of the problem and identify exposures associated with infestation in order to inform evidence-based control measures.

## Methods

**Figure 1:**
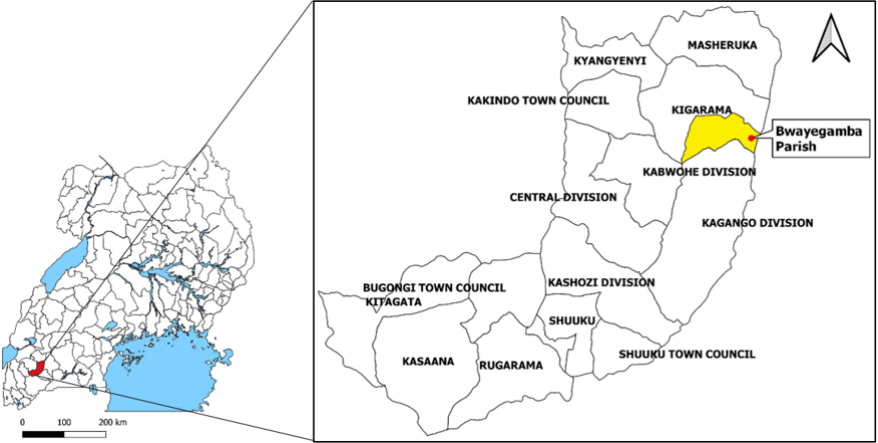
map of Sheema District showing the location of Bwayegamba Parish, Kigarama Sub-county

**Outbreak area:** Sheema District is a relatively new district in South Western Uganda that was curved out of Bushenyi District in 2010 (Figure 1). The district is administratively divided into 3 Counties, 14 sub-counties, and 41 Parishes. Kigarama, the affected sub-county comprises 7 Parishes and 97 Villages [12]. The district has a projected 2012 population of 220,300 people [13]. The main economic activities in the district include crop and livestock farming [13].

**Case definition and finding:** we defined a confirmed case as presenting with itching, pain, a burrow at the site of infestation with visible presence of *Tunga penetrans* in the skin (dark papule with a central pit) of any household member resident in Bwayegamba Parish in February 2022. We defined a suspect case as reporting of being infested with *T. penetrans*, with visible scars on the feet or any other part of the body in a period of three months before the time of interview by any resident of Bwayegamba Parish. This time period was chosen because it takes approximately 6-8 weeks from eggs hatching to the time a female sand flea grows in size and releases its eggs outside the host body [3]. We actively searched for cases in six out of 14 villages in Bwayegamba Parish known to have cases, namely: Kiyungu West, Rwakahitsi, Kikuto I, Kiyungu East, Nyamiko, and Mwengura. With the guidance of the area Local Council I (LCI) chairmen and members of village health teams (VHTs) of affected villages, we visited homes of case-households in the affected villages. We used a standardized tool to line-list cases and conducted interviews with case-patients or their parents as necessary to understand patient history and risk factors.

**Descriptive epidemiology:** we described the line-listed case-patients by person, place, and time. We constructed an epidemic curve to show the distribution of cases over the study period of November 2021 to February 2022. We computed attack rates by sex, age, and village using population projections from the Uganda Bureau of Statistics (UBOS) [12]. We drew a choropleth map to show the most affected villages in the parish. Maps were developed using Quantum Geographic Information System (QGIS) software version 3.16 with administrative shapefiles from UBOS hosted on the United Nations Office for the Coordination of Humanitarian Affairs´ (UNOCHA) Humanitarian Data Exchange (HDX) online database [14].

**Hypothesis generation interviews and environmental assessment:** we visited the homes of 12 households located in three of the six affected villages, namely; Mwengura, Kiyungu East, and Nyamiko, and assessed sanitation and hygiene by observing household compounds, surroundings and housing conditions (floors, walls), keeping animals that could act as reservoirs, and knowledge of ways to get rid of jiggers. We walked around the homes and the neighbourhoods to identify environmental conditions that are conducive to fleas. The factors that were found to affect 30% of the cases during hypothesis generation were further assessed using a case-control study design.

**Case-control studies:** to test the hypotheses, we conducted unmatched case-control studies at the household and individual level. Households with one or more cases were classified as case-households and those without any household members infested as control-households. We conducted interviews among case-households and control-households in the three most affected villages, namely: Kiyungu West, Rwakahitsi, and Kikuto I in Bwayegamba Parish. Control-households were identified within the same villages as case-households. We visited all 383 households in the three most affected villages and interviewed 278 households with an adult present at the time of the interview. Out of these, we identified 41 case-households and 237 control-households. In total, we interviewed 412 people. We collected and compared data regarding sociodemographic characteristics of cases in case-households and controls in control-households. Data regarding the case-control studies was collected using Kobo collect [15], downloaded as a Microsoft Excel file and analyzed using Epi-Info version 7.2.5.0 and STATA version 14.2. For the household case-control study, we assessed whether neighbors were infested during the study period, house walls, floor, compound, keeping animals, animal shelter, distance to water source, and ownership of a list of 15 household items, including electricity, radio, television, table, chair, sofa set, bed, cupboard, refrigerator, mobile phone, computer, bicycle, motorcycle, car, and animal drawn cart. We then used principal component analysis to predict wealth index scores premised on ownership of the above 15 household items. We used this to generate a composite household wealth index of two levels (lower and upper). We created composite binary variables for categories used to assess house walls (cracked, rough), house floors (earthen, dusty, cracked), and household compound (dirty, littered). We also assessed mitigation measures against jiggers which was defined as using a pin or needle to remove the sand flea from infested body parts immediately after it is seen. For the individual case-control study, we assessed sex, age group, education, occupation, marital status, and personal hygiene. We generated composite binary variables for age group, education, occupation, marital status, and personal hygiene (dirty feet, dirty clothes, long nails, walking barefooted). Mantel-Haensel odds ratios and p-values derived from two by two tables were used to determine the associations of exposures with jigger infestation at 95% confidence intervals.

**Ethical considerations:** the Ministry of Health, through the Director General of Health Services, gave the directive to conduct this study. We further sought administrative clearance from the District Health Office before conducting the study. We additionally obtained clearance from the office of the Director for Science, US Centers for Disease Control and Prevention (CDC), who determined that this study was in response to a public health problem and therefore non-research. All participants were asked to give verbal informed consent, and parents were asked to give consent and assent for their children before interviews. Only households with an adult present at the time of the interview were interviewed. To ensure anonymity, all information was coded with a unique identifier. Study participants were assured of confidentiality as only team members had access to the data.

## Results

**Figure 2:**
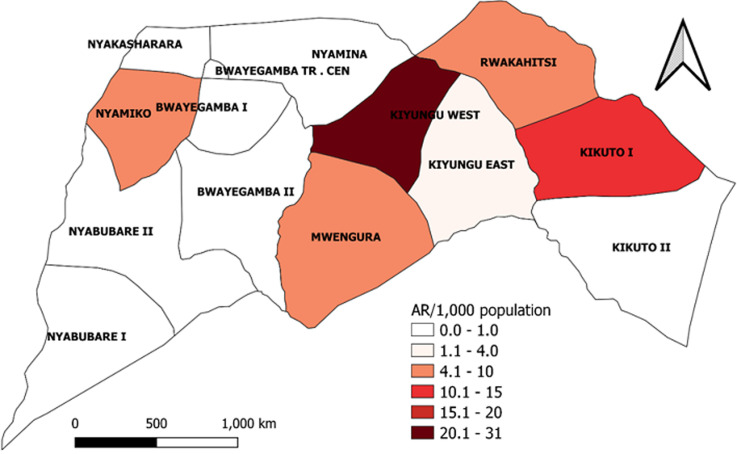
attack rates by village during a tungiasis outbreak investigation, Bwayegamba Parish, Sheema District, Uganda, November 2021-February 2022

**Figure 3:**
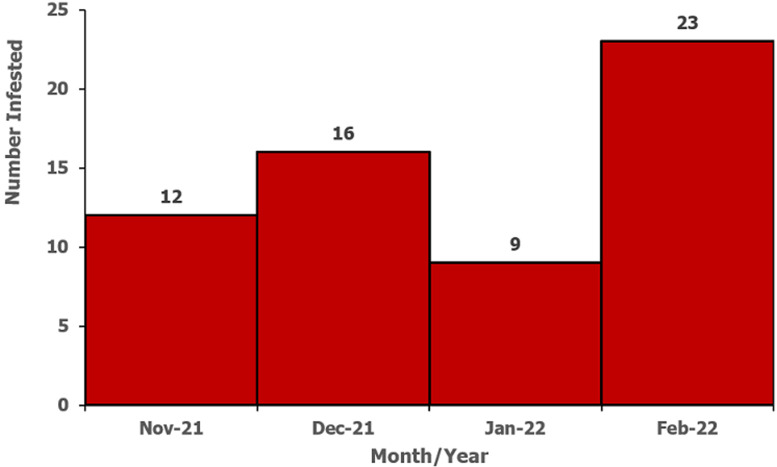
epicurve of case-patients during a tungiasis outbreak investigation, Bwayegamba Parish, Sheema District, Uganda, November 2021-February 2022

**Descriptive epidemiology:** out of 278 households visited, we identified 41 (15%) case-households. Among 412 household members, 60 (15%) were either currently infested (n=32/60, 53%) or reported ever being infested (n=28/60, 47%) in a period of three months preceding the date of interview. Males (AR/1,000 population = 13) and people in age groups 21-40 (AR/1,000 population = 16) and 41+ (AR/1,000 = 17) were most affected by jigger infestations in the six villages of Bwayegamba Parish. Village attack rates ranged from 2-31 per 1,000 population (Figure 2). The majority of participants were most recently infested in February 2022 (n=23/60) (Figure 3).

**Hypothesis generation findings:** the study was conducted during a semi-dry season. We observed poor sanitation and hygiene, characterized by littered and dirty compounds, people wearing visibly dirty clothes, having long dirty nails and walking barefooted with muddy feet. Similarly, the majority of houses had earthen floors, which may provide a sandy environment conducive to the flea. We hypothesized that factors such as household member infested in the past three months (n=10/12, 83%), household member currently infested with jiggers (n=5/12, 42%), keeping animals (n=10/12, 83%), housing animals inside main house (n=5/12, 42%), earthen floor (n=8/12, 67%), cracked floor (n=9/12, 75%), dirty floor (n=8/12, 67%), dusty floor (n=7/12, 58%), rough wall (n=8/12, 67%), cracked wall (n=6/12, 50%), littered and dirty compound (n=6/12, 50%) were associated with being a case-household. Individual factors such as having been infested for more than one month (n=4/12, 33%), knowledge of any measure for getting rid of jiggers (n=12/12, 100%), walking barefoot (n=8/12, 67%), and wearing dirty clothes (n=7/12, 58%) were associated with being a case (Table 1).

**Table 1:** hypothesis generation interview findings during a tungiasis outbreak investigation, Bwayegamba Parish, Sheema District, November 2021-February 2022

	Frequency (n)	Percentage (%)
Variable	(N=12)	
Limited knowledge of measures to get rid of jiggers	12	100
Household member infested in past 3 months	10	83
Keep animals	10	83
Walking barefooted	8	67
Earthen floor	8	67
Dirty floor	8	67
Rough wall	8	67
Cracked floor	9	75
Dirty compound	6	50
Littered compound	6	50
Cracked wall	6	50
Dirty clothes	7	58
Dusty floor	7	58
Household member currently infested	5	42
Neighbour is infested	5	42
Animals kept inside house with humans	5	42
Infested for more than a month	4	33

**Case-control studies**

**Description of case-households and control-households during an outbreak investigation of jiggers, Sheema District, Uganda, November 2021-February 2022:** compared to control-households, most case-households fell within the upper wealth quintile (n=24 (59%), p=0.059), reported practicing mitigation measures against jiggers (n=36 (88%), p=0.019), had higher proportions of cracked and rough house walls (n=31 (76%), p=0.04), littered and dirty compounds (n=20 (49%), p=0.013) and kept animals (n=25 (61%), p=0.009). Both case (n=36 (88%)) and control-households (n=176 (74%), p=0.06) had earthen, dusty and cracked floors. Most case-households (n=30 (73%)) and control households (n=156 (66%), p=0.356) kept their animals in a separate shelter outside the main house. Most case-households (n=34 (83%)) and control-households lived within 500m of a water source (n=175 (74%), p=0.214) (Table 2).

**Table 2:** household exposures vs. household infestation with jiggers during a tungiasis outbreak investigation, Bwayegamba Parish, Sheema District, November 2021 - February 2022

Household exposure (N=278)	Case-HHs* (N=41) n (%)	Control-HHs* (N=237) n (%)	ORM-H†	95% CI‡	P-value
**Wealth quintile**					
Upper	24 (59)	101 (43)	ref	0.26-1.03	
Lower	17 (41)	136 (57)	0.53		0.059
**Practice mitigation measures§**					
Yes	36 (88)	166 (70)	ref	0.11-0.82	
No	5 (12)	71 (30)	0.33		0.019
**Neighbour infested**					
No	23 (56)	164 (69)	ref	0.88-3.46	
Yes	18 (44)	73 (31)	1.75		0.099
**Wall**					
Not cracked or rough	10 (24)	98 (41)	ref	1.04-4.86	
Cracked or rough	31 (76)	139 (59)	2.18		0.04
**Floor**					
Not Earthen, dusty or cracked	5 (12)	61 (26)	ref	0.98-7.44	
Earthen, dusty or cracked	36 (88)	176 (74)	2.49		0.06
**Compound**					
Not littered or dirty	21 (51)	168 (71)	ref	1.17-4.57	
Littered or dirty	20 (49)	69 (29)	2.31		0.013
**Keeps animals**					
No	16 (39)	48 (20)	ref	0.2-0.82	
Yes	25 (61)	189 (80)	0.4		0.009
**Animal shelter**					
Outside main house	30 (73)	156 (66)	ref	0.32-1.46	
Inside main house	11 (27)	81 (34)	0.71		0.357
**Water source**					
< 500m	34 (83)	175 (74)	ref	0.23-1.34	
> 500m	7 (17)	62 (26)	0.58		0.214

* HHs: households; †ORM-H: mantel haensel odds ratio; ‡CI: confidence interval; §Practice mitigation measures: using a pin or needle to remove the sand flea from infested body parts immediately it is seen

**Description of cases and controls during an outbreak investigation of jiggers, Sheema District, Uganda, November 2021–February 2022:** compared to controls, a higher proportion of cases were male (n=34/60, 57%, p=0.003), with a mean age of 30.1(SD= +21) years. Most cases (n=38/60, 63%) and controls (n=273/352, 78%, p=0.018) fell within the 21+ age group. Compared to controls, most cases (n=31/60, 52%, p=<0.001) were unmarried and unemployed (n=33/60, 55%, p=<0.001). Compared to controls, a higher proportion of cases had poor personal hygiene (n=47/60, 78%) vs. (n=173/352, 49%, p=<0.001). Among cases, 35% (n=21/60) of them reported to have been infested with jiggers for a period of more than one month (Table 3).

**Table 3:** individual exposures vs. being infested with jiggers during a tungiasis outbreak investigation, Bwayegamba Parish, Sheema District, November 2021 - February 2022

Individual exposure (N=412)	Cases (N=60) n (%)	Controls (N=352) n (%)	ORM-H*	95% CI†	P-value
**Sex**					
Female	26 (43)	223 (63)	ref		
Male	34 (57)	129 (37)	2.26	1.3-3.96	0.003
**Age group**					
<20	22 (37)	79 (22)	2	1.1-3.57	0.018
>20	38 (63)	273 (78)	ref		
**Education level**					
Did not attend school	14 (23)	56 (16)	0.62	0.32-1.24	0.157
Attended school	46 (76)	296 (84)	ref		
**Marital status**					
Single/never married	31 (52)	93 (26)	2.97	1.69-5.22	<0.001
Married/widowed/divorced	29 (48)	259 (74)	ref		
**Occupation**					
Employed	27 (45)	70 (20)	ref		
Unemployed	33 (55)	282 (80)	3.28	1.84-5.84	<0.001
**Comorbidity**					
No	47 (78)	270 (77)	ref		
Yes	13 (22)	82 (23)	0.91	0.46-1.74	0.782
**Poor personal hygiene**					
No	13 (22)	179 (51)	ref		
Yes	47 (78)	173 (49)	3.73	1.98-7.38	<0.001

* ORM-H: mantel haensel odds ratio; †CI: confidence interval

**Factors associated with jiggers’ infestation at household level, during an outbreak, Sheema District, Uganda, November 2021–February 2022:** case-households had lower odds of practicing mitigation measures against jigger infestation (ORMH=0.33, 95% CI: 0.1-0.8) than control-households. Case-households had higher odds of living in homes with littered or dirty compounds (ORMH=2.31, 95% CI: 1.2-4.6) than control-households (Table 2).

**Factors associated with jiggers’ infestation at individual level, during an outbreak, Sheema District, Uganda, November 2021-February 2022:** cases had higher odds of being male (ORMH=2.26, 95% CI: 1.3-4.0), in the 1-20 years age group (ORMH=2, 95% CI: 1.1-3.6), unmarried (ORMH=2.97, 95% CI: 1.7-5.2), unemployed (ORMH=3.28, 95% CI: 1.8-5.8), and having poor personal hygiene (ORMH=3.73, 95% CI: 2-7.4) than controls (Table 3).

## Discussion

This investigation aimed at establishing the magnitude of tungiasis in Sheema District and identifying exposures associated with infestation in order to inform evidence-based control measures. Our results show that at household level, poorly maintained compounds were associated with infestation. At the individual level, being male, aged 1-20 years, unemployed, and having poor personal hygiene were associated with tungiasis. Whereas, practicing mitigation measures was significantly protective against infestation. At the household level, having littered or dirty compounds was significantly associated with tungiasis. This finding is similar to other studies which found that the occurrence of tungiasis was associated with littered compounds [10,16]. Poorly maintained compounds and environments around the home may attract animals such as cats, dogs, goats, and pigs which have been identified as reservoirs of the sand flea [1,17] thereby propagating the vector. The Ministry of Health, through VHTs, should continue supporting rural communities to improve sanitation by sweeping compounds and digging garbage compost pits to help keep away stray animals, which act as reservoirs for the vector. At the individual level, cases had significantly higher odds of being male. This is in agreement with a study conducted in Eastern Uganda which found males were most affected [18] and a study in Nigeria which found males having the highest number of viable tungiasis lesions [19]. This result may be explained by gender differences in personal hygiene and grooming. Females generally bathe more often than males and this may result in them noticing and removing the flea as soon as they can. Similarly, we found that poor personal hygiene such as wearing dirty clothes, having dirty feet, having long nails, and walking barefooted was significantly associated with tungiasis. This is similar to other studies that also found an association between poor personal hygiene and being infested with jiggers [10,16,20,21]. However, since our study found that the majority of households lived within 500m of a water source, lack of access to water may not account for the observed poor personal hygiene. Poor personal hygiene may therefore be due to individual or behavioral characteristics and shows a great need for continuous health education by VHTs about frequent washing and bathing, through which one may notice embedded fleas and remove them. A study conducted in Kenya found that frequent washing with soap may reduce severity of tungiasis [22] while a randomized control trial in Madagascar found that frequent wearing of shoes and using Zanzarin, a plant-based repellent, prevents infestation [23].

Furthermore, we found that more than a third of cases were infested for a period of greater than one month. Long infestation periods may point to the role of people in the propagation of the vector within their environments when the eggs are released, as modelled by a study conducted in Eastern Uganda which showed that more than seven new jigger infections could be caused by a single individual [24]. This emphasizes the need for continuous health education and awareness raising campaigns about the removal and prevention of infestation with the sand flea. We found that being in the 1-20-year age group was significantly associated with tungiasis. This finding agrees with other studies which found the highest prevalence among 1-15-year age category [19,22,25]. However, due to differences in age categories, this result should be interpreted with caution as those who are older than 15 years of age may be in a better position to notice embedded fleas and remove them compared to those younger than 15 years of age. Children are most likely to be found walking barefooted and playing in soils that are contaminated with the sand flea. Besides the pain, walking impairment, and deformed nails [5], tungiasis has other debilitating effects among children. A study conducted in Rwanda, for instance, identified tungiasis as a reason for absenteeism from school among school going children [26] while a study in Kenya found a lower quality of life among children with tungiasis [27]. Thus, implementing multipronged interventions that focus on improving household living conditions may in turn help children attain a better quality of life. In addition, being unemployed was significantly associated with tungiasis. This could be due to impairing effects of tungiasis such as pain, itching, combined with shame and stigma that prevent case-patients from seeking medical care [1,28,29]. Tackling social factors like employment and increasing income has been shown to lead to greater attainment in health outcomes [30,31]. Similarly, an increase in income implies that households may have money available to buy soap to use while bathing. Therefore, the Ministry of Health should liaise with other ministries and government departments to ensure improvement in the health and social-economic status of rural communities. On the other hand, households that practiced mitigation measures against jiggers were significantly protected against infestation. Mitigation measures, defined here as the immediate removal of the sand flea from infested body parts once it is noticed, are essential in reducing the suffering inflicted by tungiasis [1]. This agrees with other studies that found that using a pin or needle to remove the sand flea was protective against tungiasis [10]. Therefore, MOH through VHTs, should raise awareness about the immediate removal of embedded fleas to reduce the severity of tungiasis in affected villages. Lastly, the number of tungiasis cases fluctuated over the four-month study period. However, there was no way to declare an outbreak as surveillance data and reporting thresholds are lacking. Presently, there´s no direct provision for reporting tungiasis using the HMIS tools as it is lumped under “other NTDs”. MOH largely relies on media reports to identify outbreaks before responding, which may not be ideal for affected communities. Thus, we call for a change in national policy towards reporting, surveillance, funding, and management of tungiasis as one of the important NTDs as recognized by the Sustainability Plan for Neglected Tropical Diseases Control Program 2020-2025 [9]. The Ministry of Health should integrate tungiasis as part of HMIS reporting enabling monitoring of interventions and prompt responses.

**Study limitations:** the main limitation of this study is that recall and social desirability bias raise doubts about the use of self-reported data [32,33]. The suspect case definition required participants to recall whether they had been infested three months before the day of interview. Such memory questions are likely to introduce recall bias as well as social desirability bias for participants or households that did not currently have an infested member. Additionally, our suspect case definition largely relied on participants´ local knowledge of what jigger infestation is. We counteracted this by using the local term (“*emira*”) for jigger infestations in the Runyankore language, which is spoken in Sheema District. This being a case-control study, we were unable to determine causality. However, we identified exposures that increased the odds of infestation with jiggers in Sheema District.

## Conclusion

Males, those who are unemployed, those in the age group 1-20 year and those who have poor personal hygiene were significantly associated with tungiasis in this outbreak. Households with littered or dirty compounds were significantly associated with infestation, whereas those that practiced mitigation measures against jiggers were protected against infestation. This calls for designing targeted interventions towards affected groups with improvements in household living conditions. There´s a need for continuous health education and awareness raising on the prevention of jigger infestations in rural communities. This can be done through existing structures such as VHTs in order to reach and help greatly affected individuals, especially those that have been infested for long periods. Accordingly, the Ministry of Health should fast track the distinct inclusion of tungiasis in HMIS reporting of neglected tropical diseases in order to avail surveillance data needed to identify future outbreaks.

**Funding:** this project was funded by the President’s Emergency Plan for AIDS Relief (PEPFAR) through the United States Centers for Disease Control and Prevention (US CDC) to the Uganda Public Health Fellowship Program (PHFP), Ministry of Health (MOH) through Makerere University School of Public Health (MakSPH). The funder had no role in the design of the study, collection of data, analysis, or decision to publish the work. Its contents are solely the responsibility of the authors and do not necessarily represent the official views of PEPFAR, US CDC, MaKSPH, PHFP, and MOH.
